# Automated imaging cytometry reveals dysplastic indices of colonic serrated adenomas

**DOI:** 10.2144/fsoa-2019-0111

**Published:** 2020-02-21

**Authors:** Nicholas S Samel, Qin Huang, Hiroshi Mashimo

**Affiliations:** 1 VA Boston Healthcare System, West Roxbury, MA 02132, USA; 2 VA Boston Healthcare System, Harvard Medical School, West Roxbury, MA 02132, USA

**Keywords:** colon polyps, dysplasia, Feulgen stain, image cytometry, serrated adenoma

## Abstract

**Aim:**

Left-sided colonic serrated adenomas (L-SAs) were evaluated for aneuploidy using automated imaging cytometry to quantify DNA content and compared with normal colonic tissues (NCT), tubular adenomas (TA), left-sided hyperplastic polyps (L-HP) and adenocarcinomas.

**Materials & methods:**

We used standard paraffin-embedded Feulgen-stained tissue sections.

**Results:**

The mean DNA index (DI) of NCT was 0.95, L-HP was 1.08, TA was 1.22, L-SA was 1.11 and adenocarcinomas was 1.46. DI of L-SA was statistically higher than that of NCT, but not statistically different from L-HP.

**Conclusion:**

This study demonstrates that DIs correlate with the described neoplastic progression of L-SA, TA and L-SA compared with NCT and suggests that L-SA may be involved in a chromosome instability pathway of neoplastic progression.

## Lay abstract

Colon cancer remains a deadly disease, with a significant burden of illness to patients and healthcare systems. While most precursor lesions will not necessarily produce cancers, they vary in histology and potential for neoplastic progression. Aneuploidy or abnormal chromosomal content of a cell is considered a marker for chromosomal instability and neoplastic progression. However, conventional methods of assessment can be laborious, costly and may even underestimate its malignant potential if the lesion is focal, small and surrounded by normal stromal cells in the sampled tissue. We used a nuclear stain to detect and quantify aneuploidy on conventionally prepared colonic precancerous histological slides and in particular assessed serrated and hyperplastic polyps of the left colon. When compared with normal tissues, we determined that there was aneuploidy in these lesions, which supports the underappreciated assumption that these lesions manifest chromosomal instability.

Colorectal cancer (CRC) is the third most common cancer diagnosed in both men and women in USA and is the third leading cause of cancer-related deaths in USA [1,2]. Since the progression from an identifiable precancerous lesion to CRC is often slow and the prognosis for patients identified at earlier stages are greatly improved [2,3], the early detection of colonic neoplasias is important. The current standard for CRC screening is by colonoscopy, which involves biopsies or resections sent to pathologists [3,4]. Although improved endoscopic imaging such as endocytomicroscopy [5], confocal microscopy [6] and optical coherence tomography in concert with artificial intelligence [7] are promising and may even preclude the need for excisional biopsies, the present pathologic evaluation of neoplastic lesions are largely qualitative and fraught with inter- and intra-observer disagreement among pathologists regarding their diagnoses [8,9]. Thus, a quantitative assessment of colorectal neoplasias is desirable.

Aneuploidy – defined as a deviation from the normal number of chromosomes in a cell as a result of whole or segmental chromosome loss or gain – is a prominent phenotype in CRC and has been considered a driver of this cancer [10–12]. Aneuploidy can be a result of many factors, including chromosome segmentation defects at the mitotic checkpoint and inactivating mutations in DNA damage repair genes [13]. The heterogeneous nature of CRCs origin, specifically within terms of its genetic instability, provides both a challenge in diagnosis and an opportunity for risk stratification [14]. Genetic instability can occur in three different forms in CRC: CpG island methylation phenotype, microsatellite instability (MSI) resulting from defective repair of mismatched bases and chromosomal instability (CIN) leading to aneuploidy [15].

CpG island methylation phenotype is associated with the silencing of tumor suppressor and DNA repair genes. It is associated with the right colon, mucinous features, MSI, poor tumor differentiation, high *BRAF* mutation rates and the female population [14]. MSI tends to arise in the right colon, is associated with a slightly better prognosis and responds differently to chemotherapy; thus, its treatment is specialized [16]. CIN is the most common pathway, representing 70–85% of CRCs [15]; these mutations can involve the *APC*, *KRAS* and *TP53* genes and altered regulation of the Wnt/β-catenin pathway leading to microsatellite stable CRCs displaying aneuploidy [17]. Since the varying genetic instabilities of CRCs could call for specialized treatments, quantitative evaluation of DNA content as a reflection of aneuploidy in biopsies and resections of suspect lesions would have clinical utility.

There are currently two main ways of examining DNA content in colonic tissue. Flow cytometry (FC), as demonstrated for Barrett’s Esophagus [18], involves disruption of an entire sample for single cell suspension and may miss focal lesions due to localized aneuploidy being diluted by a majority of normal diploid cells. For example, one study showed standard FC to miss 29% of aneuploidy cases in Barrett’s adenocarcinoma [19]. Image cytometry, however, is a useful tool for visualizing cellular distribution *in situ*, allowing for more precise, localized information while preserving spatial information [20]. While image cytometry has been typically carried out using the semi-automated CAS 200 (Becton Dickenson, CA, USA) system, an automated cellular imaging system (ACIS) has been shown to be more sensitive and less time consuming than CAS 200 for determining DNA ploidy [21]. ACIS can produce high-fidelity histograms, which have been shown to provide more details that are potentially important predictors of progression in gastrointestinal cancers [22]. However, ACIS has yet to be evaluated on colonic tissue, as its pioneer work was conducted in the esophagus in the study of Barrett’s Esophagus. A weakness of ACIS has been its suboptimal technique in delineating cells and differentiating nonoverlapping nuclei (termed Watershed Segmentation [19]) and correcting its errors are often time consuming. Our technique, automated imaging cytometry (AIC), uses an operator in lieu of Watershed Segmentation, produces high-fidelity histograms and operates at the same rate as ACIS.

AIC has the potential to evaluate localized aneuploidy in colon samples, such as in serrated adenomas (relatively flat neoplasias found in the colon). Serrated adenomas have the potential to develop into adenocarcinoma via an alternative pathway, called the serrated pathway, which is responsible for as much as 30% of CRCs [23]. Although the pathway of serrated adenomas to adenocarcinomas is unclear, a small subset of serrated adenomas is known to harbor *TP53* mutations and Wnt/β-catenin pathway activation, suggesting the presence of a CIN-like pathway [17]. A recent study by Choi *et al.* demonstrated using FC that a subset of serrated adenomas demonstrated aneuploidy, shedding some light onto the pathway of their carcinogenesis [17]. Hyperplastic polyps have generally been understood to be a benign subset of serrated adenomas, although whether they also possess a neoplastic pathway is not well understood.

Given the frequent disagreement among pathologists regarding morphological diagnosis of hyperplastic and sessile serrated adenomas in the right colon [24,25], we chose to focus on hyperplastic and serrated adenomas taken only from the left colon, where diagnoses are more definitive [26,27]. Of note, DNA methylation and mismatch repair are more commonly found in hyperplastic polyps of the right colon [28], a fact possibly behind the diagnostic confusion or overlap in the right colon. Moreover, *BRAF* mutations are largely found in the right colon [29] and would therefore not be likely to display aneuploidy, while *KRAS*-mutated polyps are typically left-sided [30]. We chose to obtain only left-sided serrated adenomas to assess whether these harbored aneuploidy and potentially *KRAS* mutations, as described by others using FC [17].

Serrated adenomas have not been evaluated for aneuploidy using AIC and their DNA content has not been quantitatively measured alongside various types of colonic neoplasias and polyps. In this study, we used AIC to investigate aneuploidy across tubular adenoma, left-sided serrated adenoma, left-sided hyperplasia and adenocarcinoma, with the goal of assessing genetic instability across a variety of colonic neoplastic conditions and paying special attention to the relative DNA content displayed by serrated adenomas.

## Materials & methods

### Tissue acquisition

Random paraffin-embedded tissue blocks spanning from 2004 to 2018 were obtained for analysis from the VA Boston Healthcare System in MA, USA, including normal colonic tissue (NCT) (normal colonic mucosal biopsies obtained during screening colonoscopies, not specified with respect to location), tubular adenoma (TA), left-sided serrated adenoma (L-SA), left-sided hyperplastic polyps (L-HP) and adenocarcinoma (AC; not specified with respect to location). The patient age range was 46–85 years old, with a mean age of 71 years old (all males). All samples obtained were less than 1 cm in diameter. No patients underwent neoadjuvant treatment, had history of prior chemotherapy or represented recurrences. The same patient in five cases has provided synchronous or metachronous lesions of nonmalignant tissue samples for this study. The pathological assessments were made based per tissue block, each with unique polyps and independent of patient identity. The study was reviewed and approved by the Institutional Review Board and Research Committee of the VA Boston Healthcare System.

### Histology & Feulgen stain

Tissue blocks were cut at 5 μm in thickness with hematoxylin and eosin (H&E; Ventana, AZ, USA) staining carried out on the samples according to a standard protocol. The slide of the adjacent section was separately Feulgen stained (Scytek Laboratories, UT, USA) to quantify nuclear DNA content [21] per the manufacturer’s instructions. After staining, the original pathological reading was confirmed and regions of interest (ROIs) were marked on each H&E slide by a specialized gastrointestinal pathologist (QH). Each H&E ROI was transcribed on the corresponding Feulgen-stained slide for analysis.

### Determination of DNA index

Slides were scanned onto a computer using an Olympus VS120 (Olympus; Tokyo, Japan) at 40× magnification. Using ImageJ, an operator delineated 30 lymphocyte control cells in each sample, followed by 150–200 epithelial cells [31]. Care was taken to ensure that the selected cells were nonoverlapping. While ImageJ was used to acquire nuclei (control lymphocyte cells and relevant glandular mucosal cells) in each ROI, images were analyzed using MATLAB in grayscale to obtain an integral (sum of all pixels) of the -10log (pixel value) for each pixel in the nucleus, which represented the integrated optical density (IOD). The IOD correlates with the DNA content and morphological features of the cell, such as its size, shape, contour, granularity and chromatin texture of the nucleus [19,22].

**Figure 1.**
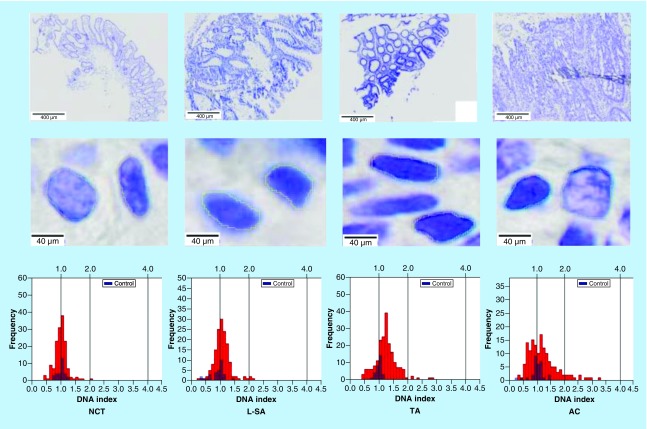
Examples of encircled nuclei of Feulgen-stained epithelial cells to derive DNA index histograms for normal colonic tissue, left-sided serrated adenoma, tubular adenoma and adenocarcinoma samples. A line representing DNA index of 1 is derived from the modal integrated optical density from encircling nuclei of lymphocytes on the same slide. The scale bars on the upper images represent about 400 μm and the scale bars on the lower images represent about 40 μm. AC: Adenocarcinoma; L-SA: Left-sided serrated adenoma; NCT: Normal colonic tissue; TA: Tubular adenoma.

In each sample, the modal IOD of the lymphocyte controls was assigned a DNA index (DI) of 1 to be an internal diploid (2N) control, which served as a reference for the experimental epithelial cells as per a previously described protocol [21]. For each sample, high-fidelity histograms (Figure 1) were generated to map DI versus number of cells, with DI defined as the ratio of epithelial IOD to control IOD. For each sample, the peak histogram DI value was considered as representative of that sample. Then, we determined the mean DI and standard deviation (SD) of the cell population for each study category. Additionally, statistical differences of each neoplasia with respect to NCT and L-SAs were evaluated using a one-tailed unpaired t-test with significance established at p < 0.05.

## Results

### Distinct DIs demonstrated for colonic tissues

Examples of encircled nuclei on single-slide Feulgen-stained samples of normal and various colonic polyps and their corresponding histograms are shown in Figure 1. Results for average DIs for each condition are summarized in Table 1. We determined NCT and L-HP to be diploid, with 33.3% of L-HPs displaying aneuploidy. TA exhibited mild aneuploidy and L-SA also showed mild aneuploidy. Lastly, AC showed moderate aneuploidy (Table 1).

**Table 1.** Mean DNA index, aneuploidy rating and fraction of samples with aneuploidy for each condition.

Condition	Mean DNA index (range; standard deviation)	Ploidy	Fraction of samples with aneuploidy
NCT	0.95 (0.81–1.05; 0.08)	Diploid	0/10 (0%)
L-HP	1.08 (0.81–1.30; 0.14)	Diploid	3/9 (33.3%)
TA	1.22 (1.02–1.52; 0.17)	Mild aneuploidy	3/8 (37.5%)
L-SA	1.11 (0.95–1.42; 0.15)	Mild aneuploidy	7/12 (58.3%)
AC	1.46 (1.20–2.04; 0.27)	Moderate aneuploidy	10/10 (100%)

AC: Adenocarcinoma; L-HP: Left-sided hyperplastic polyp; L-SA: Left-sided serrated adenoma; NCT: Normal colonic tissue; TA: Tubular adenoma.

### Increased DI of colonic adenomas compared with normal colonic tissue

**Figure 2.**
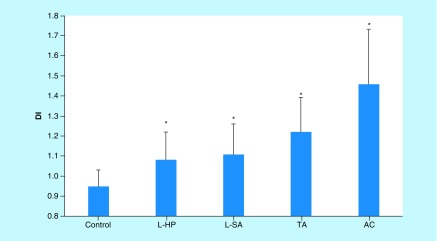
DNA index comparison of normal colonic tissue, left-sided hyperplastic polyp, left-sided serrated adenoma, tubular adenoma and adenocarcinoma. *Indicates statistical significance in DI from normal colonic tissue. AC: Adenocarcinoma; DI: DNA index; L-HP: Left-sided hyperplastic polyp; L-SA: Left-sided serrated adenoma; TA: Tubular adenoma.

Results from t-tests comparing the DIs of the various tissue types are summarized in Table 2. DIs of L-HP, L-SA, TA and AC were significantly different from the DI value of NCT (p < 0.05; Figure 2). With respect to L-HP, all other tissue types had significant DI differences except for L-SA. With respect to L-SA, L-HP and TA had insignificant differences (p > 0.05), while NCT and AC had significant differences in DI (p = 0.02, 0.00, respectively). The DI of TA was significantly different from all tissue types (p < 0.05) except for L-SA (p = 0.07) and the DI of AC was significantly different from all tissue types (p < 0.05).

**Table 2.** One-tailed t-test comparisons for normal colonic tissue and polyps.

Condition	NCT	L-HP	L-SA	TA	AC
NCT	X	0.02^†^	0.02^†^	0.00^†^	0.00^†^
L-HP	X	X	0.37	0.03^†^	0.00^†^
L-SA	X	X	X	0.07	0.00^†^
TA	X	X	X	X	0.01^†^
AC	X	X	X	X	X

† Indicates statistical significance at an α of 0.05.

AC: Adenocarcinoma; L-HP: Left-sided hyperplastic polyp; L-SA: Left-sided serrated adenoma; NCT: Normal colonic tissue; TA: Tubular adenoma.

## Discussion

This study shows that aneuploidy can be semi-quantified in colonic serrated adenomas using AIC, a simple procedure using open-source software (ImageJ and MATLAB), AIC DI correlates with the described neoplastic progression of colonic adenomas to adenocarcinomas, tubular and serrated adenomas possess a similar but significant increase in DI when compared with NCT and the aneuploidy displayed by serrated adenomas might suggest a CIN pathway.

**Figure 3.**
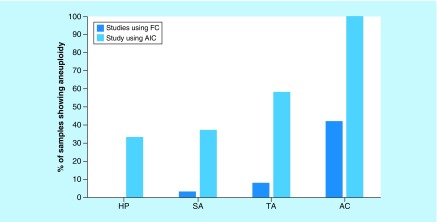
Comparing flow cytometry (from literature) and automated imaging cytometry in terms of the resulting percent of samples with aneuploidy for each type of colonic lesion. AC: Adenocarcinoma; AIC: Automated imaging cytometry; FC: Flow cytometry; HP: Hyperplastic polyp; SA: Serrated adenoma; TA: Tubular adenoma.

Aneuploidy can be quantified in the colon using AIC. Our results show aneuploidy in 33.3% of L-HPs, 37.5% of L-SAs, 58.3% of TAs and 100% of ACs (Table 1). Previous studies using FC have found aneuploidy in 0% of hyperplastic polyps [32], 3.3% of traditional serrated adenomas without high grade dysplasia [17], 7.4–8.1% of TAs [33,34] and 42% of colorectal AC [35]. This is shown graphically below (Figure 3). Our DI results coincide with the described malignancy risks of these polyps and show a greater (100%) aneuploidy in known adenocarcinomas compared with FC (Figure 3). The potential clinical utility of AIC will require prospective studies.

Statistical analysis demonstrates that the polyps (L-HP, L-SA, TA and AC) have generally higher DIs than NCT (Figure 3). However, while L-HP did not demonstrate sufficiently high DI to meet the threshold for mild aneuploidy (DI > 1.1), its unexpectedly significantly higher DI compared with NCT demonstrates increase in DNA content. While L-HP has generally been accepted to have very low or minimal progression to adenocarcinoma, one retrospective study suggests that L-HP indeed bears some risk for neoplastic progression [36]. The increase in DI for L-SA is also consistent with increasing appreciation of their malignant potential. The relationship between HP and serrated adenomas is poorly understood, but given their similar DIs, our results suggest the involvement of the CIN pathway. Additionally, assessing for CIN in serrated or hyperplastic polyposis syndrome (SPS, HPS) samples could shed light onto the pathway of neoplastic progression for serrated polyposis syndrome and HPS and *KRAS* mutations have been implicated in both [37]. Nevertheless, larger follow-up studies are required to assess whether the patients with L-HP and L-SA demonstrating elevated DI indeed have higher neoplastic risks compared with those with normal DI. Thus, further care is advised in detecting L-HP and L-SA, which can be challenging given their flat shape and ability to camouflage with the surrounding tissue, often with overlying mucin.

Serrated adenomas are identifiable pathologically by the ‘saw-toothed’ appearance in their crypt epithelia, while HPs are generally considered to be benign and distinct from serrated adenomas. However, numerous studies [38–40] have suggested that in the proximal colon, this benign feature of HPs should be challenged in favor of suspecting the serrated pathway. The pathway of serrated carcinogenesis and whether it involves CIN remain poorly understood [41]. Our result of L-HP and L-SA harboring aneuploidy with similar DIs is a possible reflection of their chromosomal instability. In this regard, serrated adenomas have been described to have aneuploidy as a result of the CIN pathway [13], *KRAS* mutations in L-SAs [42], Wnt pathway activation [43] and p53 mutations [44]. Thus, AIC has the potential to identify a subset of HPs and serrated adenomas, which has a greater neoplastic potential to serve as biomarkers to supplement traditional pathology. FC appears to miss this subset of aneuploid cells, possibly owing to dilution by predominant diploid cells in the samples.

It is also conceivable that in more advanced neoplasia, the variability of DI among cells could increase. AIC demonstrates not only increased DI, but a higher variability in DNA content within the tissue when compared with that of normal tissue and other neoplastic tissues, as reflected in the higher overall SD between AC and normal samples (Figure 1). For example, the variability of DI between cells is greatest in AC (i.e., 0.27, Table 1). Additionally, the SD for L-HP, TA and L-SA are similar (Table 1), while the SDs for normal colonic tissue is the lowest at 0.08. This wider variation in DI may also reflect a higher nuclear pleomorphism and well-known tumor heterogenicity characteristically associated with AC. Thus, besides increasing DI with malignancy potential, there is an increased SD of the DI. Further studies of SDs per sample could be investigated to verify whether this spread in DI could be used as a biomarker for relative malignancy.

While AIC demonstrated that the DI of AC is significantly higher than that of other polyps or NCT, more work is needed to investigate its ability to differentiate among precancer lesions in the colon. While pathology can be subject to inter- and intra-observer variability, AIC may provide a more objective, nuanced and semiquantitative means of evaluating precancerous conditions in the colon. In the future, digital image information and analysis is conducive to improved, fast and automated computer-aided diagnosis, even at remote sites where expert pathological interpretation may be scarce. Larger prospective studies are needed to evaluate AIC’s ability to identify patients at risk for cancer progression and include the pathological analysis of other known biological markers, particularly for the CIN pathway. Our single-center pilot study was indeed limited by tissue availability. While this limitation bears risk for the study being underpowered, we believe these data serve as a useful foothold for future studies seeking to use this technique to address a broader population-based sensitivity and specificity in the future. Additionally, while we concentrated on serrated adenomas and TA, further studies are required to explore adenomas with villous and tubulovillous features, which have been described to have greater malignancy potential. Pure villous adenomas are rare, but future studies are required to assess whether DI correlates with known greater malignancy risk. Furthermore, a subgroup analysis comparing the dysplasia indices of right- versus left-sided adenocarcinomas would be of interest and would require greater powered studies in the future.

## Conclusion

In conclusion, AIC has the ability to quantify aneuploidy across various colonic polyps and neoplasias. In the colon, it suggested that that some hyperplastic polyps harbored aneuploidy. It also suggested that serrated adenomas may progress to cancer through aneuploidy via a chromosomal instability pathway. Additionally, AIC demonstrated that tubular and serrated adenomas have intermediate DNA indices when compared with normal mucosa and adenocarcinoma.

## Future perspective

With the rise of artificial intelligence, analysis of such digitized images as described in this study may allow characterization of computer-recognizable markers and plays an increasingly supportive role in the understanding and recognition of cancer pathology. This study posits the utility of aneuploidy as a neoplastic marker and additionally reveals the potential for the CIN pathway in L-HP and L-SA samples. Further research with more data points into aneuploidy of colon cancer progression, specifically for L-HP and L-SA, is necessary to support this claim. However, its confirmation would have diagnostic implications, as aneuploidy can serve as a potential marker for neoplastic progression of hyperplastic polyps, a class of atypia whose pathological disambiguation could lead to a reduction in CRC incidence.

**Summary points**

- Automated imaging cytometry can assess for aneuploidy in various colonic polyps.
- Paraffin-embedded tissue blocks of normal colonic tissues, tubular adenomas, left-sided serrated adenomas, left-sided hyperplastic polyps (L-HP) and adenocarcinomas were sectioned, stained with Feulgen dye and analyzed for nuclear DNA content using automated image cytometry.
- DNA index (DI) was calculated by obtaining the mean integrated optical density of the nuclei of the different epithelial cells and expressing these as a ratio to the mean integrated optical density of lymphocyte nuclei on adenocarcinomas slide.
- Left-sided serrated adenomas showed increased DI compared with DI of normal colonic tissues and similar increase in DI as that of tubular adenomas.
- A subset of L-HP also showed elevated DIs, suggesting that, although traditionally considered benign, some L-HP manifest aneuploidy and may have increased potential for neoplastic progression.
- Aneuploidy evidenced in serrated adenomas suggests the involvement of the chromosomal instability pathway in its neoplastic progression.
